# Macrofungal Diversity and Funga in Two Forest Types of Motuo Ecosystems, Southwest China

**DOI:** 10.3390/jof12070533

**Published:** 2026-07-19

**Authors:** An-Hong Zhu, Zhi Qu, Si-Yu Ma, Zhong-Xin Sun, Rong-Shen Li, Hai-Xia Ma

**Affiliations:** 1 Haikou Key Laboratory for Conservation and Utilization of Edible and Medicinal Fungal Germplasm Resources, Hainan Key Laboratory of Tropical Microbe Resources, Institute of Tropical Bioscience and Biotechnology, Chinese Academy of Tropical Agricultural Sciences, Haikou 571101, China; 18289679317@163.com (A.-H.Z.); quzhi@itbb.org.cn (Z.Q.); masiyu@mails.jlau.edu.cn (S.-Y.M.); 15947908270@163.com (Z.-X.S.); 18469348046@163.com (R.-S.L.); 2 Coconut Research Institute, Chinese Academy of Tropical Agricultural Sciences, Wenchang 571339, China; 3 School of Ecology and Nature Conservation, Beijing Forestry University, Beijing 100083, China; 4 College of Plant Protection, Jilin Agricultural University, Changchun 130118, China; 5 Chongzuo Key Laboratory for Conservation and Utilization of Edible and Medicinal Fungal Germplasm Resources, Guangxi Research Institute, Chinese Academy of Tropical Agricultural Sciences, Chongzuo 532100, China

**Keywords:** ecology, macrofungal diversity, species composition, southeast Xizang

## Abstract

The study on macrofungi diversity in Motuo county, Xizang Autonomous Region, was conducted from 2021 to 2025, and 2286 vouchered fungal collections were obtained from local forest ecosystems. A total of 534 species, representing two phyla, eight classes, 28 orders, 96 families, and 241 genera were identified according to the morphological characters and molecular evidence. The orders Agaricales (174), Polyporales (91), Xylariales (74) and Russulales (69) were the dominant orders, collectively accounting for 76.40% of the total species listed. Fifteen dominant families with more 10 species each were identified, namely Russulaceae (58), Polyporaceae (37), Xylariaceae (34), Hypoxylaceae (30), Boletaceae (17), Psathyrellaceae (13), Mycenaceae (12), Agaricaceae (11), Hymenochaetaceae (11), Auriculariaceae (11), Stromphariaceae (11), Marasmiaceae (10), Omphalotaceae (10), Steccherinaceae (10), and Pleurotaceae (10), which occupied approximately 53.37% of the total species. Additionally, 21 dominant genera with more than five species each were recorded, including *Russula* (27), *Xylaria* (25), *Hypoxylon* (17), *Lactarius* (16), *Lactifluus* (15), and other 16 genera (101), accounting for around 37.64% of all species. We further compared species composition, distribution patterns, dominant taxa and trophic modes of macrofungi between tropical monsoon forest and evergreen broad-leaved forest. A total of 439 species (82.21%) were endemic to a single forest type, while only 95 species (17.79%) were shared by the two forest habitats. The evergreen broad-leaved forest exhibited slightly higher macrofungal species richness than the tropical monsoon forest in Motuo County. Moreover, it harbored a higher proportion of ectomycorrhizal fungi (25.32% vs. 14.06%) and a lower proportion of saprotrophic fungi (71.52% vs. 83.71%). This study fills the research gap in systematic macrofungal diversity investigation in Motuo County, and provides fundamental data for revealing the evolutionary mechanisms of macrofungi on the Qinghai-Xizang Plateau margin and clarifying the biodiversity formation process across the Himalayan region.

## 1. Introduction

Fungi are one of the most diverse group of organisms on Earth; the recent estimate of the total fungal diversity ranged from 2.2 to 3.8 million [1], of which more than 150,000 taxa have been scientifically described [2,3,4,5,6]. Among these species, macrofungi that produce visible fruiting bodies listed more than 51899 species with a main worldwide distribution in MTSEM (http://www.nmdc.cn/macrofungi/; accessed 20 May 2025). Macrofungi, as wood degraders, pathogens and ectomycorrhizal fungi play a crucial role in forest development and the maintenance of the ecological balance [7,8,9,10,11,12]. In addition, many macrofungal taxa are of great interest due to their economic significance. Numerous species are renowned as wild edible mushrooms and medicinal fungi in various regions around the world for thousands of years [13,14,15,16,17,18,19]. Therefore, they are important strategic biological resources for scientific significance and application value. To protect and utilize these resources, it is important to recognize and understand the species diversity of these resources as much as possible [20,21,22].

Worldwide systematic surveys of macrofungi diversity have being conduncted for the past two decades, including North America [23,24], Africa [25], Europe [26,27,28] and Asia [29,30,31,32,33,34,35,36]. In China, investigations and statistics on the macrofungal diversity have focused on some cities or counties [37,38,39,40,41], famous reserves [42,43,44,45], national forest park [46,47,48,49], typical vegetation zone [50,51,52,53,54,55], and important groups of macrofungi [56,57,58,59]. However, there have been insufficient investigations of macrofungi for their remote locations, and systematic research on diversity has not yet been carried out.

Motuo County is located in the southeast of Xizang Autonomous Region, China, in the hinterland of the eastern Himalayas, and is recognized as one of the global hotspots of biodiversity [60,61,62]. The vegetation zones are characterized by obviously vertical structures including tropical monsoon forest, evergreen broad-leaved forest, conifer-broadleaf mixed forest, subalpine dark coniferous forest, and alpine shrub meadow [63]. The complex geographical conditions, unique three-dimensional climates and diverse vegetations contribute to the abundant resources of macrofungi. Due to its remote location, harsh and changeable climate, and frequent geological disasters, there has been insufficient investigation of macrofungi in Motuo, and systematic research on diversity has not yet been carried out. Until 1983, four macrofungi, namely *Cordyceps racemosa* Berk., *Exobasidium pieridis* Henn., *Grifola frondosa* (Dicks.) Gray (syn. *Polyporus frondosus* (Dicks.) Fr.), and *Pleurotus limpidus* (Fr.) P. Karst., were first documented during the First Tibetan Plateau Scientific Expedition [64]. In 1982 and 1983, Xiaolan Mao and colleagues performed macrofungal field surveys in the Nmjagbarwa Mountain area, and subsequently published a monograph recording 143 macrofungal species from Motuo County [65]. In recent years, a batch of new macrofungal species and new distribution records have been continuously reported from Motuo County [66,67,68,69,70,71,72,73,74,75,76,77]. Nevertheless, all the aforementioned studies lack systematic, long-term and integrated field investigations of Motuo macrofungi. Furthermore, research focusing on macrofungal diversity, ecological adaptation and community assembly across distinct forest habitats remains scarce in southeastern Xizang, and Motuo County remains one of the macrofungal research hotspots with the most insufficient published datasets.

To clarify the macrofungal diversity and species composition across two important forest types in Motuo comprehensively, we conducted systematic field surveys in tropical monsoon forests and evergreen broad-leaved forests from 2021 to 2025. This study presents the first dataset from long-term macrofungal diversity monitoring of the core forest ecosystems in Motuo. Meanwhile, we clarified the macrofungal species composition and dominant taxa (families and genera), analyzed the trophic modes of identified fungal species, and elucidated the distribution discrepancies of macrofungi between the two vegetation types. In addition, we further compared the macrofungal diversity patterns of Motuo with those of adjacent forested regions.

## 2. Materials and Methods

### 2.1. Sampling Area and Strategy

The geographical location of Motuo is between 93°46′–96°05′ E and 27°34′–29°56′ N with an altitude of 154–7782 m (http://www.motuo.gov.cn/; accessed 30 June 2026). Motuo’s climate is a typical three-dimensional climate zone with coexistence of tropical, subtropical, temperate and frigid zone. This study was carried out in two forest ecosystems of Motuo County; the main vegetation types investigated are tropical monsoon forest and evergreen broad-leaved forest. The investigated area is characterized by the low-mountain tropical humid climate zone and the mountain subtropical humid climate zone with a mean annual precipitation of 2000–2500 mm with peaks from March–October (http://www.motuo.gov.cn/; accessed 30 June 2026). Our investigation included eight investigated sites (ca. 2.5 ha per site), four of which were located in evergreen broad-leaved forest and four in tropical monsoon forest. Detailed site information regarding location, elevation, coordinate, and dominant trees is listed in Table S1.

Macrofungal samples were collected through a simple random sampling method from different sites only during the rainy season (May–October) of 2021 to 2025. Seven field trips were made: the first in September to October 2021, the second and third in July and October respectively in 2023, the fourth and fifth in June and September in 2024, and the other two in May and August in 2025. Field sampling could not be implemented in 2022 due to COVID-19 pandemic control restrictions. Sampling was conducted from May to October to cover the full rainy season in five years, aiming to capture the maximum possible macrofungal species diversity. The sampling procedure was the same for the two investigated forests in order to obtain comparable data useful to analyze how diversity varied along the studied vegetation type. Surveys of each forest ecosystem were conducted at comparable frequencies and sampling intensities by the same investigators (4–5 persons). Each site was sampled within a narrow time window (1–2 days per survey) to minimize temporal variation between sampling events.

### 2.2. Sample Collection and Specimen Preparation

Fresh fruiting bodies of macrofungi growing on all substrates were collected by systematic survey from each site. The specimens were photographed in situ by using a Canon G16 camera (Canon Corporation, Tokyo, Japan) and their fresh macroscopic, organoleptic characteristics of fruiting bodies (e.g., odour and colour), and trophic modes were recorded. The context or site tissue (1–2 g) of the fresh specimens was stored in a sealed bag with silica gel for DNA extraction. The fresh specimens were dried with an electronic portable dryer (Foshan, China) at 40 °C for 24 h, and then the dried specimens were labeled and stored in an ultra-low freezer at –80 °C for 1 week. The prepared specimens were deposited in the Fungarium of the Institute of Tropical Bioscience and Biotechnology, Chinese Academy of Tropical Agricultural Sciences (FCATAS).

### 2.3. Species Identification

The specimens were identified on the basis of macro- and micro-morphological features, using guide books, monographs and related references [78,79,80,81,82,83,84,85,86,87,88,89,90,91,92,93]. All collected fungal specimens were subjected to internal transcribed spacer (ITS) sequencing. Total genomic DNA of studied samples was extracted using a cetyltrimethylammonium bromide (CTAB) rapid extraction kit (Aidlab Biotechnologies, Beijing, China). ITS region was amplified with primer pair ITS5 and ITS4 [94]. Species identification combined morphological characteristics with BLAST comparisons against NCBI databases, with specimens showing over 98% ITS sequence similarity assigned to the corresponding species. Formal phylogenetic analyses were not performed in this study. Most current names of species and their taxonomy status were checked according to Index Fungorum (https://www.speciesfungorum.org/; accessed 14 July 2026) [95] and related references. The classification of geographical elements mainly followed distribution data from Global Biodiversity Information Facility (GBIF, https://www.gbif.org). No bulk occurrence dataset with a dedicated download DOI was generated in this research.

### 2.4. Statistical Analyses

Species richness in the two forests was estimated using Chao2 and Jacknife2 estimators [96,97,98]. We applied the Sorensen similarity index to compare the two investigated forest stands by analyzing the species composition (SC), which reflects the proportion of shared macrofungal taxa in the total number of taxa of specific habitat pairs. For demonstrating the species composition of macrofungi, Origin 9.0 software, Microfost Office Excel 2007, and SRplot tools were used to produce the Barplot, Pieplot, Venn diagram, and Voronoi diagram.

## 3. Results

### 3.1. Species Composition

**Figure 1:**
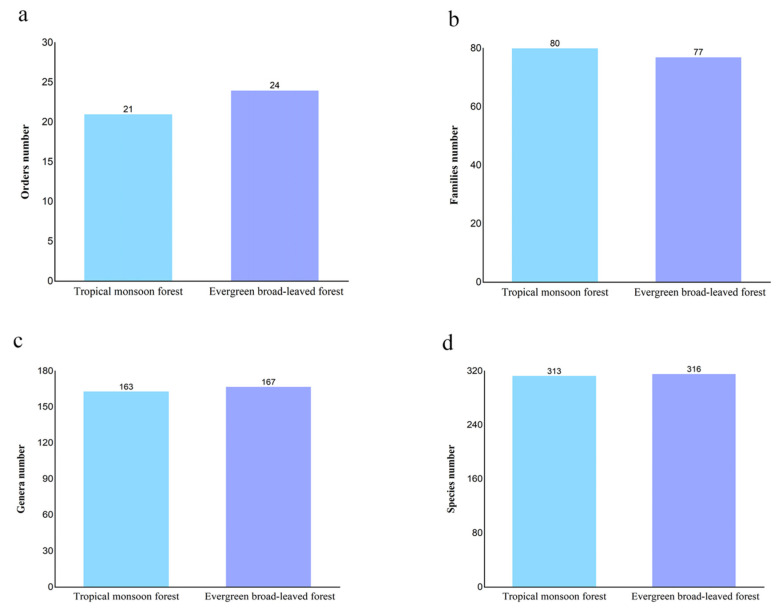
The number of orders (**a**), families (**b**), genera (**c**), and species (**d**) found in Tropical monsoon forest (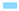) and evergreen broad-leaved forest (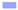).

In this study, a total of 2286 specimens were collected from our investigation in the past five years in Motuo County. The taxonomic checklist with taxonomic classification and collection information was provided for each species (Table S2). A total of 534 macrofungal species were identified, belonging to two phyla, eight classes, 28 orders, 96 families and 241 genera (Figure S1). Basidiomycota was the dominant phylum, comprising three classes, 17 orders, 71 families, 189 genera and 414 species, whereas Ascomycota included five classes, 11 orders, 25 families, 52 genera and 120 species. A total of 313 species from 21 orders, 80 families and 163 genera were recorded in tropical monsoon forest, while 316 species spanning 24 orders, 77 families and 167 genera were found in evergreen broad-leaved forest (Table 1 and Figure 1). We used the Chao2 and Jackknife2 estimators to predict the overall macrofungal species richness of the studied communities. The Chao2 estimator predicted 1201 species, nearly double the observed species number. The Jackknife2 estimator produced a relatively conservative estimate of 1065 species.

### 3.2. Species Richness

**Figure 2:**
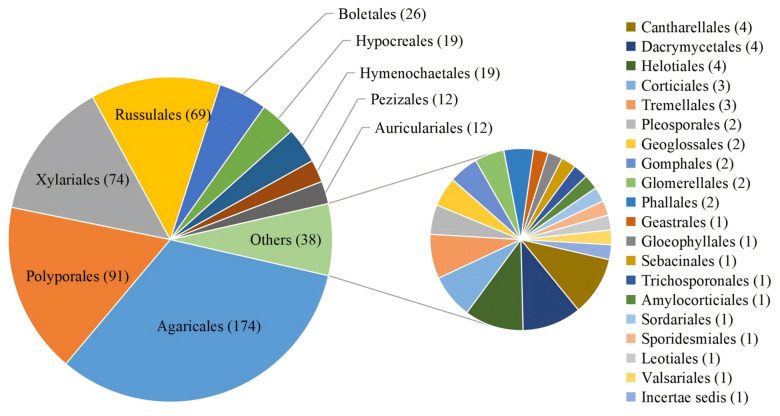
Taxonomic composition of macrofungi species at the order level in Motuo County.

Among the 534 species in Motuo County, the Agaricales was the most diverse order with 174 species accounting for 32.58%, followed by Polyporales (91/534) accounting for 17.04%, Xylariales (74/534) accounting for 13.86%, and Russulales (69/534) accounting for 12.92%, and the other five orders with more than 10 species, viz. Boletales (26), Hypocreales (19), Hymenochaetales (19), Pezizales (12), and Auriculariales (12), accounting for 4.87%, 3.56%, and 2.25% respectively, while the remaining nineteen orders accounted for a mere 7.11% (Figure 2). The species proportion of orders varied in the two forests. The Agaricales displayed a higher species richness in the two forests, ranging from 29.11% in evergreen broad-leaved forest to 33.55% in tropica monsoon forest. The other two dominant orders, Polyporales and Xylariales, had a higher species richness in tropica monsoon forest compared with evergreen broad-leaved forest, while the order Russulales has a higher species richness in evergreen broad-leaved forest.

**Figure 3:**
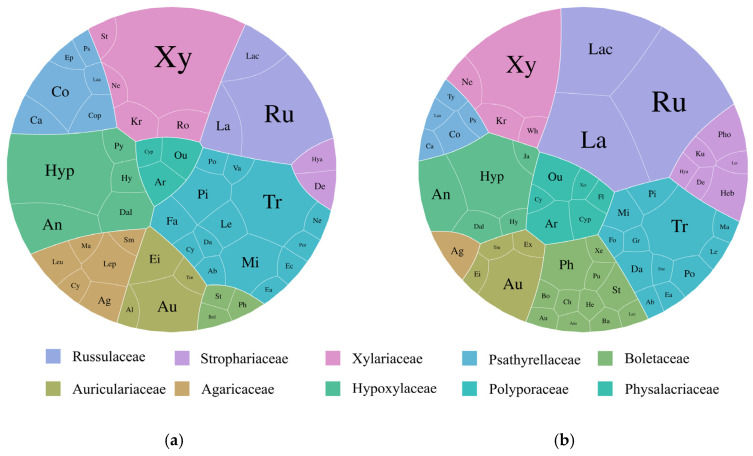
Voronoi diagram of species proportions of dominant family in (**a**) Tropical monsoon forest and (**b**) Evergreen broad-leaved forest. Colors represent the different families. Genera names are represented by an abbreviation following Table S3.

At the family level, the Russulaceae was the most diverse family with 58 species accounting for 10.86% of all the species in Motuo, followed by the Polyporaceae, Xylariaceae and Hypoxylaceae with 37, 34, and 30 species accounting for 6.93%, 6.37%, and 5.62% respectively, as well as 375 species that belonged to 92 families and accounted for the remaining 70.22%. The Russulaceae exhibited a higher species richness in evergreen broad-leaved forest (46 species) compared with tropic monsoon forest (18 species), while the Polyporaceae, Xylariaceae, and Hypoxylaceae displayed a higher species richness in tropic monsoon forest (28, 24, and 22 species) compared with evergreen broad-leaved forest (22, 19 and 18 species), respectively (Table 1, Figure 3).

### 3.3. Endemic Species Versus Shared Species

**Figure 4:**
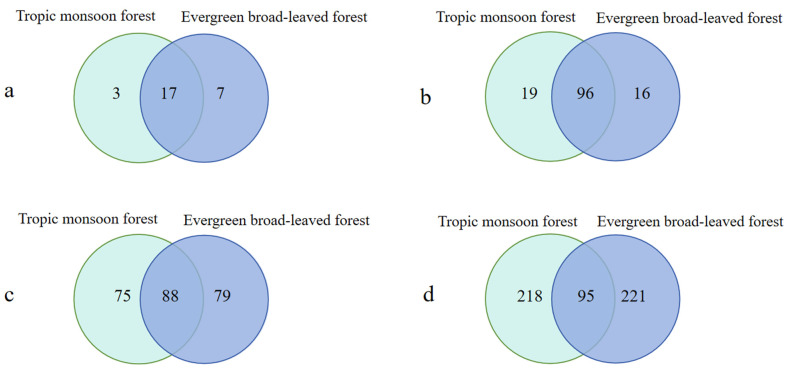
Venn diagram of species diversity in Tropical monsoon forest and Evergreen broad-leaved forest in (**a**) orders, (**b**) families, (**c**) genera, and (**d**) species.

Seventeen orders were distributed in the two forests (60.71%; 17/28), and three endemic orders were distributed in tropic monsoon forest, seven endemic orders were found in evergreen broad-leaved forest (Figure 4a). Among the 96 families, 61 (63.54%) were distributed in two forests, with 19 endemic families in tropic monsoon forest, and 16 endemic families in evergreen broad-leaved forest (Figure 4b). Of the 241 genera, 88 were distributed in two forests accounting for 36.51%, and 75 and 79 were endemic genera accounting for 31.12% and 32.78% in tropic monsoon forest and evergreen broad-leaved forest, respectively (Figure 4c). Only 95 species, accounting for 17.79% (95/534), were shared in two forests, while 439 species were endemic accounting for 82.21% (Figure 4d). Evergreen broad-leaved forest and tropic monsoon forest had a high SC value up to 0.30.

### 3.4. Trophic Modes

**Figure 5:**
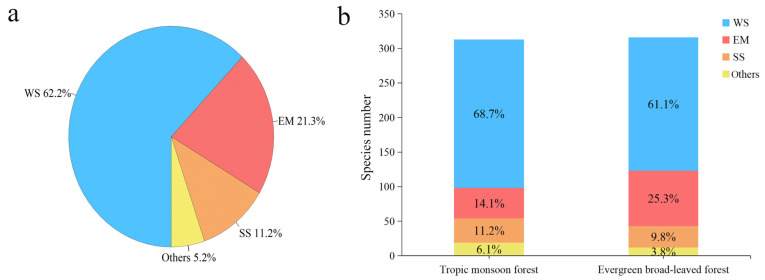
Macrofungi species in the different nutritional modes. (**a**) Proportions of different nutritional nodes in all species in this study. (**b**) Proportions of species in Tropical monsoon forest and Evergreen broad-leaved forest. Color legend shows the different nutritional modes.

Among the 534 species, wood-decaying fungi (WS) were dominant in the Motuo County, consisting of 332 species which accounts for 62.17% of all species (Figure 5a). Ectomycorrhizal fungi (EM) were the second most abundant with 114 species accounting for 21.35%, followed by soil-saprotroph fungi (SS) with 60 species accounting for 11.24%. The remaining six nutritional fungi, viz. bamboo-decaying fungi (BS, 8 species), endophyte-insect pathogen (EI, 7 species), litter-saprotroph fungi (LS, 5 species), termite-symbiont fungi (TS, 3 species), fungicolous fungi (FF, 3 species), and fruit-saprotroph fungi (FS, 2 species), with a total 28 species observed in the two forests.

In tropica monsoon forest, the numbers of wood-decaying, ectomycorrhizal, soil-saprotroph, bamboo/litter/fruit saprotroph, and other nutritional mode species (EI and TS) were 215, 44, 35, 12, and 7, respectively, and they accounted for 68.69%, 14.06%, 11.18%, 3.83%, and 2.24% of all species found in this forest (Figure 5b). In evergreen broad-leaved forest, the numbers of wood-decaying, ectomycorrhizal, soil saprotroph, bamboo/litter/fruit saprotroph, and other nutritional mode species were 193, 80, 31, 5, and 7, respectively, and they accounted for 61.08%, 25.32%, 9.81%, 1.58%, and 2.21% of all species found in this forest (Figure 5b). The proportions of wood-decaying and ectomycorrhizal fungi in the two forests are significantly different. The wood-decaying fungi displayed a higher species richness in tropica monsoon forest, while ectomycorrhizal fungi had a higher species richness in evergreen broad-leaved forest.

### 3.5. Biogeographical Patterns of Macrofungi

The present study shows that eight geographic distribution patterns of macrofungi at thg species level were found in the Motuo County. The elements of cosmopolitan (130), northern temperate (115), pantropical (34), palaeotropical (15), tropic Asian-tropical Australian (42), tropica Asian-tropic Australian-tropic American (48), east Asian (95), and Chinese endemic (55) accounted for 24.34%, 21.54%, 6.37%, 2.81%, 7.87%, 9.0%, 17.79%, and 10.30%, respectively. The tropical elements, including pantropical, palaeotropical, tropic Asian-tropical Australian, and tropica Asian-tropic Australian-tropic American elements, displayed a slightly higher proportion with 139 species accounting for 26.03%, followed by cosmopolitan, northern temperate, east Asian and endemic elements.

**Figure 6:**
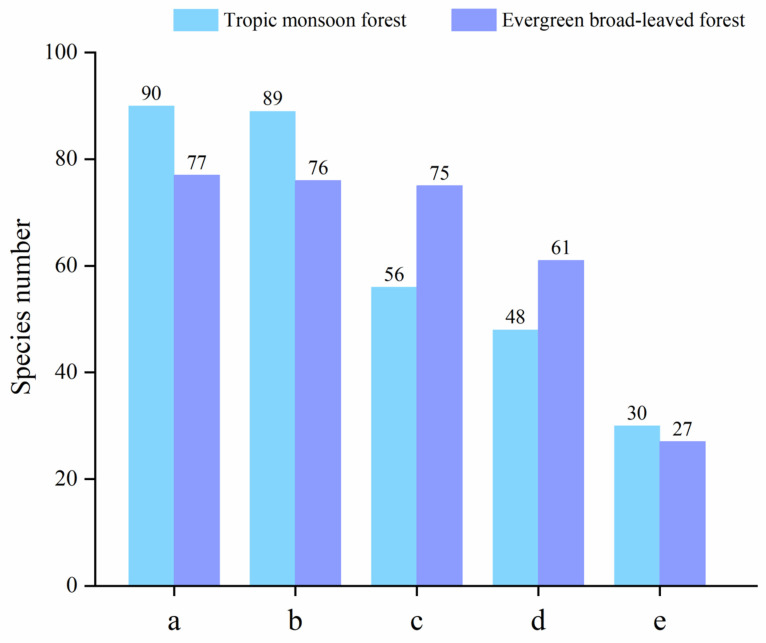
Geographic distribution patterns of species in Tropical monsoon forest and Evergreen broad-leaved forest. (a) Cosmopolitan elements, (b) Tropical elements, (c) Northern temperate elements, (d) East Asian elements, and (e) Endemic elements.

In tropica monsoon forest, the cosmopolitan and the tropical elements were dominant with 90 and 89 species accounting for 28.75% and 28.43% of all species, with the tropical elements consisting of pantropical with 20 species, palaeotropical with l7 species, tropic Asian-tropical Australian with 28 species, and tropica Asian-tropic Australian-tropic American with 34 species respectively. Northern temperate, east Asian, and endemic elements with 56, 48 and 30 species were found accounting for 17.89%, 15.34% and 9.59% respectively (Figure 6). In evergreen broad-leaved forest, the numbers of northern temperate (77), cosmopolitan (76), and tropical elements (75) were not significantly different, accounting for 24.37%, 24.05%, and 23.73% of all species respectively. East Asian elements with 61 species and endemic elements with 27 were found accounting for 19.30% and 8.55% respectively.

## 4. Discussion

In recent years, macrofungal species diversity across diverse habitats has been extensively investigated worldwide, and numerous fungal species have been formally documented globally [24,25,28,36,99,100,101,102,103,104,105,106]. Most recently, Liu et al. (2025) reported 192 macrofungal species in this region, including 188 Basidiomycota species and four Ascomycota species [77]. However, systematic and comprehensive surveys on macrofungal diversity have long been lacking in Motuo County, southeastern Xizang Autonomous Region. Here, we conducted five-year systematic field surveys (2021–2025) on fungal assemblages across two important forest ecosystems in Motuo County. In total, 534 macrofungal taxa were recovered, which were assigned to eight classes, 28 orders, 96 families and 241 genera. Nonetheless, the recorded taxa are still far from reflecting the full magnitude of macrofungal diversity of this region. The Chao2 species richness estimator predicted that the actual macrofungal richness of the local communities is nearly twice the observed number, with a predicted value of 1201 species, while the Jackknife2 estimator provided a relatively conservative prediction of 1065 species. Owing to prevalent sampling biases, both estimators likely underestimate the true regional species richness. As documented in previous studies [107,108,109], richness estimators can be affected by limitations in ecological field sampling. Only sustained supplementary sampling can enable comprehensive documentation of macrofungal diversity within these ecosystems.

The two forests of Motuo County exhibited higher macrofungal orders and species number compared than adjacent regions [30,110,111,112]. In the present study, macrofungal taxa assigned to 28 orders and one order of incertae sedis within Basidiomycota and Ascomycota were identified from the two forest ecosystems. Comparative data showed that Wang et al. (2015) recorded 16 orders (11 Basidiomycota and five Ascomycota orders) in forests of Southeastern Xizang [110], Pradhan et al. (2016) reported 18 orders (13 Basdiomycota and five Ascomycota orders) in the Eastern Himalayas [30], while Debnath et al. (2020) documented 16 orders in Triipura, India [111]. Our results confirmed that the forest habitats of Motuo possess higher macrofungal order and species diversity than most fugal-rich forest areas reported previously [105,113,114]. Motuo forests are characterized by extraordinarily high vascular plant diversity, which provides sufficient and heterogeneous substrates in humid tropical forest habitats. Such diversified substrate resources further promote the differentiation of macrofungal assemblages and shape distinct macrofungal biodiversity patterns [115].

The macrofungal species composition revealed that Agaricales is dominated by both tropica monsoon forest and evergreen broad-leaved forest of Motuo County, and this dominance pattern has been widely reported across various forest zones worldwide [36,37,101,103,111,116,117,118,119,120,121,122]. The dominance of Agaricales can be explained by its versatile trophic strategies, including substrate decomposition of dead branches and fallen litter, ectomycorrhizal symbiosis and parasitic lifestyles. In this study, Agaricales (174 taxa) exhibited multiple trophic guilds, consisting of 89 wood-saprotrophic taxa, 50 soil-saprotrophic taxa, 27 ectomycorrhizal taxa, five litter-saprotrophic taxa and three termite-symbiotic taxa. By contrast, Polyporales (91 WS taxa), Xylariales (66 WS taxa, 6 BS taxa, 2 FS taxa) and Russulales (8 WS taxa, 61 EM taxa) possessed far simpler trophic structures. Overall, Agaricales harbored more diversified trophic modes and broader host substrate spectra, which improves environmental adaptability and further drives its higher species richness.

The present study identified Russulaceae as the most dominant family, which is consistent with previous macrofungal surveys in multiple protected forest areas [27,30,113,114,123,124]. Members of Russulaceae represent a core ectomycorrhizal group, playing crucial symbiotic roles in tropical, subtropical, and temperate forest biomes [125,126,127,128,129]. Beyond Russulaceae, other dominant ectomycorrhizal fungal families detected in this survey included Boletaceae (17 taxa) and Amanitaceae (eight taxa), followed by Cortinariaceae and Inocybaceae (five taxa each), Paxillaceae (four taxa), as well as Hydnangiaceae, Hebelomataceae, and Hydnaceae (three taxa each). The remaining ectomycorrhizal families contained only one or two taxa. These ectomycorrhizal fungi are widely documented to form symbiotic associations with diverse host tree families, including Fabaceae, Fagaceae, Betulaceae, Dipterocarpaceae, Ericaceae, Orchidaceae, Pinaceae, and Rosaceae [130,131,132,133,134,135,136].

Polyporaceae constituted the second most species-rich family with 37 taxa, followed by Xylariaceae (34 taxa) and Hypoxylaceae (30 taxa), all of which are typical wood and litter saprotrophs. A total of 404 saprotrophic taxa were recorded in this study, accounting for 75.66% of the 534 macrofungal taxa documented in Motuo County. The diversity and abundance of saprotrophic fungi in the two Motuo forest types are primarily shaped by habitat characteristics, substrate availability, and relative air humidity, which aligns with the conclusions of previous fungal ecological studies [137,138]. Nevertheless, a contrasting pattern was reported by Rakić et al. (2022) [28], who demonstrated that saprotrophic fungal abundance was mainly driven by soil moisture and air temperature, while air humidity exerted no significant regulatory effect on saprotrophic fungal communities.

Our results revealed that saprotrophic fungi occupied a considerably higher proportion than ectomycorrhizal fungi (75.66% vs. 21.35%), with the species number of saprotrophs being 3.5 times that of ectomycorrhizal taxa. Saprotrophic fungi are generally more sensitive to short-term climatic fluctuations than ectomycorrhizal fungi, which enables them to maintain higher diversity under variable weather conditions [139]. Furthermore, well-conserved and healthy forest ecosystems typically harbor a higher proportion of mycorrhizal fungi, which can serve as a key ecological indicator of forest stability [140,141,142,143]. Previous studies have proposed that a predominance of saprotrophic fungi over ectomycorrhizal counterparts usually indicates forest disturbance and habitat degradation, which is generally driven by anthropogenic interference, silvicultural operations, and livestock grazing [144,145,146]. Collectively, various forest habitats in Motuo offer suitable environments for diverse macrofungal assemblages, and sustain remarkably abundant saprotrophic fungal communities. Abundant substrate supplies, high tree species heterogeneity and unique local microclimates jointly determine the distribution patterns and biodiversity of macrofungi in this area.

Consistent with our hypothesis, the two forest types in Motuo County harbored distinctly differentiated macrofungal communities. Both forest ecosystems exhibited a predominance of saprotrophic fungi over mycorrhizal and parasitic fungi. Nevertheless, the dominant fungal families differed markedly between the two vegetation types. The evergreen broad-leaved forest was predominated by Russulaceae (46 taxa), accounting for 14.56% of its total macrofungal species. In contrast, Polyporaceae was the most species-rich family (28 taxa, 8.95%) in the tropical monsoon forest, while Russulaceae ranked fourth with only 18 species (5.75%). Additionally, the evergreen broad-leaved forest supported a significantly higher richness of mycorrhizal fungi (80 taxa) than the tropical monsoon forest (44 taxa). The coupled high richness of Russulales and abundant ectomycorrhizal fungi in evergreen broad-leaved forests, as well as the co-occurrence of high Polyporales richness and dominant wood-saprotrophic fungi in tropical monsoon forests, could indicate that environmental filtering regulates not only macrofungal species diversity but also the functional traits and ecological functions of local fungal assemblages.

Among all recorded macrofungal taxa, only 95 species were shared between the two forest types, accounting for 17.8% (95/534) of the total species, whereas habitat-endemic species collectively reached 82.2%. Our findings demonstrated that tropical monsoon forests and evergreen broad-leaved forests harbor highly heterogeneous macrofungal assemblages with pronounced divergence in species composition, trophic modes, and biogeographic affinities. This distinct community differentiation occurred even though the two forest habitats are geographically adjacent and possess comparable species richness (313 vs. 316 taxa). Such community divergence can be primarily attributed to the divergent habitat heterogeneity shaped by contrasting vegetation structures between the two forest types [147,148,149]. High regional precipitation and complex habitat heterogeneity generate diverse microhabitats, enabling diverse saprotrophic fungi to acquire specific substrates for growth and survival [25]. Furthermore, varied vegetation composition modulates the quantity and quality of organic matter inputs, which further shapes the assembly of saprotrophic macrofungal communities [150]. The dominance of wood- and litter-decomposing macrofungi observed in this study is a typical characteristic of tropical forest ecosystems, which is consistent with previous global fungal ecological investigations [137]. Ectomycorrhizal taxa, mainly belonging to Russulaceae, Boletaceae, and Amanitaceae, were significantly enriched in evergreen broad-leaved forests dominated by Fagaceae and Pinaceae host trees, compared with tropical monsoon forests. The two forest types also differ greatly in floristic composition: tropical monsoon forests are dominated by purely tropical floral elements, while evergreen broad-leaved forests integrate both tropical and temperate floral components. Located in the Eastern Himalayan biodiversity hotspot, Motuo County represents a unique convergence zone of tropical and temperate biotas. The exceptionally high macrofungal diversity in this region is comprehensively shaped by heterogeneous forest habitats and its pivotal biogeographic position as a floristic transition zone.

Although the present study provides fundamental baseline data on macrofungal diversity, ecological interpretation remains preliminary. Constrained by sampling frequency and sampling intensity, we mainly focus on describing diversity and fungal characteristics. Targeted investigations adopting standardized sampling designs combined with multivariate statistical analyses will help disentangle the environmental factors driving macrofungal distribution across this region.

## 5. Conclusions

This study systematically investigated the biodiversity and functional distribution of macrofungi in tropical monsoon forests and evergreen broad-leaved forests of Motuo County, Xizang Autonomous Region, China. Five years of sampling across eight sites uncovered remarkable high macrofungal diversity, with Basidiomycota as the predominant group and Ascomycota accounted for nearly one quarter of all recorded taxa. Species belonging to Agaricales and Polyporales constituted the dominant components of the local macrofungal community. Comparative analysis revealed that evergreen broad-leaved forests harbored a higher richness of ectomycorrhizal fungi but a lower proportion of saprotrophic fungi relative to tropical monsoon forests. This systematic dataset fills the regional research gap and provides fundamental baseline information for understanding the adaptive evolution of macrofungi on the Qinghai-Xizang Plateau margin and elucidating the biodiversity formation mechanisms across the Himalayan hotspot. Nevertheless, the present inventory still cannot fully reflect the complete macrofungal diversity of Motuo’s forest ecosystems. The insufficient species inventory restricts our comprehensive interpretation of macrofungal ecological functions, including their roles in forest regeneration, fungal diversity hotspot evaluation, and community responses to environmental fluctuations. Therefore, future investigations could expand sampling coverage and survey efforts to unravel how habitat and climatic factors shape the assembly and distribution of macrofungal communities within this distinctive transitional zone. Meanwhile, one limitation of this work is that it focuses on macrofungal species inventory rather than community ecology exploration. No a priori ecological hypotheses were established, and analyses of community assembly, β-diversity and habitat filtering were not performed. Future studies with targeted ecological hypotheses and long-term sampling will help explore the assembly rules of local fungal communities.

## Figures and Tables

**Table 1:** Composition of macrofungi in tropical monsoon forest (TMF) and evergreen broad-leaved forest (EBF).

Order	Family	Genera		Species	
		TMF	EBF	TMF	EBF
Pleosporales	Astrosphaeriellaceae	0	1	0	1
	Pyrenochaetopsidaceae	1	0	1	0
Valsariales	Valsariaceae	1	0	1	0
Geoglossales	Geoglossaceae	1	1	1	1
Helotiales	Chlorociboriaceae	0	1	0	1
	Helotiaceae	2	1	2	1
Leotiales	Leotiaceae	0	1	0	1
Pezizales	Pezizaceae	1	1	1	1
	Pyronemataceae	2	3	2	3
	Sarcoscyphaceae	2	2	5	2
	Sarcosomataceae	1	0	1	0
Glomerellales	Glomerellaceae	1	0	1	0
	Plectosphaerellaceae	1	0	1	0
Hypocreales	Bionectriaceae	1	1	1	1
	Clavicipitaceae	0	1	0	1
	Cordycipitaceae	2	2	3	2
	Hypocreaceae	1	3	1	7
	Nectriaceae	2	2	2	2
	Ophiocordycipitaceae	1	1	1	1
Incertae sedis	Incertae sedis	1	0	1	0
Sporidesmiales	Sporidesmiaceae	1	0	1	0
Sordariales	Lasiosphaeriaceae	0	1	0	1
Xylariales	Diatrypaceae	4	1	5	4
	Graphostromataceae	1	1	1	1
	Hypoxylaceae	5	5	21	18
	Xylariaceae	5	4	24	18
Agaricales	Agaricaceae	6	1	10	2
	Amanitaceae	1	1	5	3
	Bolbitiaceae	1	1	1	2
	Clavariaceae	2	2	2	3
	Cortinariaceae	1	1	2	3
	Crepidotaceae	1	1	3	2
	Cyphellaceae	1	1	1	1
	Entolomataceae	1	1	3	4
	Galeropsidaceae	1	1	1	1
	Hydnangiaceae	1	1	2	2
	Hygrophoraceae	0	2	0	3
	Hymenogastraceae	2	3	4	5
	Incertae sedis	5	6	7	8
	Inocybaceae	1	1	4	1
	Lycoperdaceae	2	2	2	2
	Lyophyllaceae	1	0	3	0
	Marasmiaceae	2	1	8	3
	Mycenaceae	5	5	7	5
	Omphalotaceae	3	2	7	4
	Physalacriaceae	3	6	5	11
	Pleurotaceae	3	3	5	7
	Pluteaceae	1	0	6	0
	Psathyrellaceae	6	5	11	6
	Pseudoclitocybaceae	1	0	1	0
	Pterulaceae	1	0	1	0
	Radulomycetaceae	1	1	1	1
	Schizophyllaceae	1	1	1	1
	Strophariaceae	2	6	2	10
	Tricholomataceae	0	1	0	1
	Tubariaceae	0	1	0	1
Amylocorticiales	Amylocorticiaceae	0	1	0	1
Auriculariales	Auriculariaceae	4	4	10	8
	Incertae sedis	0	1	0	1
Boletales	Boletaceae	3	11	3	15
	Gyroporaceae	1	0	1	0
	Paxillaceae	1	1	4	1
	Sclerodermataceae	1	0	1	0
	Suillaceae	0	1	0	1
	Tapinellaceae	0	2	0	2
Cantharellales	Hydnaceae	2	2	2	2
Corticiales	Corticiaceae	1	0	1	0
	Punctulariaceae	1	1	1	1
Geastrales	Geastraceae	1	0	1	0
Gloeophyllales	Gloeophyllaceae	0	1	0	1
Gomphales	Gomphaceae	0	1	0	2
Hymenochaetales	Hirschioporaceae	1	1	1	1
	Hymenochaetaceae	5	3	8	6
	Hyphodontiaceae	0	1	0	1
	Incertae sedis	0	1	0	1
	Schizoporaceae	2	1	5	1
Phallales	Phallaceae	2	1	2	1
Polyporales	Dacryobolaceae	1	0	1	0
	Fomitopsidaceae	1	2	1	3
	Ganodermataceae	1	1	6	5
	Hyphodermataceae	1	0	1	0
	Incertae sedis	0	1	0	1
	Incrustoporiaceae	1	1	1	1
	Irpicaceae	3	2	3	2
	Laetiporaceae	1	0	1	0
	Meripilaceae	1	1	3	1
	Meruliaceae	2	3	2	3
	Panaceae	2	1	6	2
	Phanerochaetaceae	3	3	3	3
	Podoscyphaceae	2	1	2	2
	Polyporaceae	14	12	28	22
	Steccherinaceae	4	2	7	5
Russulales	Auriscalpiaceae	1	0	1	0
	Hericiaceae	1	0	1	0
	Peniophoraceae	0	1	0	1
	Russulaceae	3	3	18	46
	Stereaceae	1	4	2	7
Sebacinales	Sebacinaceae	0	1	0	1
Dacrymycetales	Dacrymycetaceae	1	2	2	3
Tremellales	Tremellaceae	1	1	2	2
Trichosporonales	Trichosporonaceae	0	1	0	1

## Data Availability

All original data generated in this research are included within the article and its Supplementary Materials.

## Supplementary Materials

The following supporting information can be downloaded at: https://www.mdpi.com/article/10.3390/jof12070533/s1, Figure S1: Taxonomic distribution of macrofungi in Motuo County at phyla, class, order, family and genus levels; Table S1: Basic site information at each study forest; Table S2: The taxonomic checklist of macrofungi from Motuo County; Table S3: The species proportions of domiant family in two forests.
