# Supplementary material for: Macrofungal Diversity and Funga in Two Forest Types of Motuo Ecosystems, Southwest China
Source: J Fungi (Basel). 2026 Jul 19;12(7):533. doi: 10.3390/jof12070533 (PMC13412882; doi:10.3390/jof12070533)
Supplement: Supplementary file 1 [file jof-12-00533-s001.zip › Figure S1.pdf]

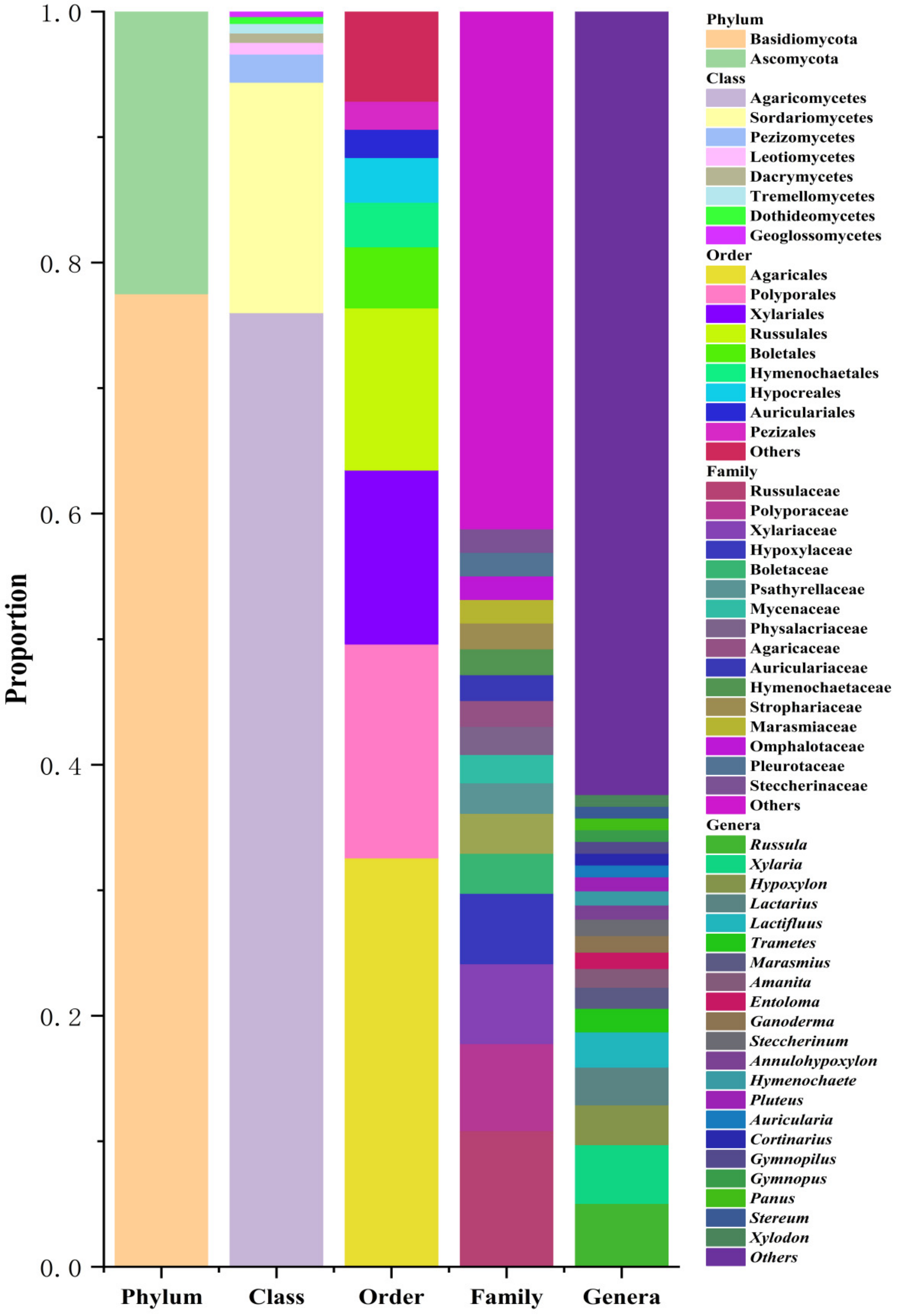

Figure S1 Taxonomic distribution of macrofungi in Motuo County at phyla, class, order, family and genus levels. All phyla and class taxa, nine order taxa with the most species, and domiant taxa of family and genus are shown. The rest taxa at order, family and genus levels are classified as "others".
