# Supplementary material for: Macrofungal Diversity and Funga in Two Forest Types of Motuo Ecosystems, Southwest China
Source: J Fungi (Basel). 2026 Jul 19;12(7):533. doi: 10.3390/jof12070533 (PMC13412882; doi:10.3390/jof12070533)
Supplement: Supplementary file 1 [file jof-12-00533-s001.zip › Table S1.pdf]

Table S1. Basic site information at each study forest, including location, study site, geographic coordinates, elevation, and dominant tree species.

Forest type	tropic monsoon forest				evergreen broad-leaved forest			
Location	Motuo Town	Beigeng Township	Beigeng Township	Beigeng Township	Motuo Town	Gandeng Township	Gandeng Township	Gandeng Township
Study site	The mountain	Buqun Lake	Jiangxin Village	Didong Village	Renqingbeng Temple	Gelin Village	Deergong Village	Gandeng Village
Geographic coordinates	29°19′ N 95°20′ E	29°15′ N 95°12″ E	29° 13′ N 95° 08′ E	29°12′ N 95°05′ E	29°18′ N 95°20′ E	29°13′ N 95°10′ E	29°11′ N 95°09′ E	29°10′ N 95°08′ E
Elevation rang (m)	700-1100	1000-1080	650-800	550-720	1150-2020	1450-1600	1370-1450	1600-1760
Dominant trees	*Altingia excelsa* Noronha (Altingiaceae), *Terminalia myriocarpa* Van Heurck & Müll. Arg. (Combretaceae), *Alnus nepalensis* D. Don (Betulaceae), *Mallotus tenuifolius* Pax (Euphorbiaceae), *Saurauia tristyla* DC. (Actinidiaceae), *Ficus semicordata* Buch.-Ham. ex Sm.				*Quercus oxyodon* Miq. (Fagaceae), *Quercus lamellosa* Sm. (Fagaceae) *Alnus nepalensis* D. Don (Betulaceae), *Pyrenaria khasianaR*. N. Paul (Theaceae), *Pinus bhutanica* Grierson, D.G.Long & C.N.Page (Pinaceae)			
